# Differential Proteomic Analysis of *Arabidopsis thaliana* Genotypes Exhibiting Resistance or Susceptibility to the Insect Herbivore, *Plutella xylostella*

**DOI:** 10.1371/journal.pone.0010103

**Published:** 2010-04-08

**Authors:** Richard M. Collins, Muhammed Afzal, Deborah A. Ward, Mark C. Prescott, Steven M. Sait, Huw H. Rees, A. Brian Tomsett

**Affiliations:** 1 School of Biological Sciences, The University of Liverpool, Liverpool, United Kingdom; 2 Institute of Integrative and Comparative Biology, University of Leeds, Leeds, United Kingdom; Cairo University, Egypt

## Abstract

A proteomic study was conducted to investigate physiological factors affecting feeding behaviour by larvae of the insect, *Plutella xylostella*, on herbivore-susceptible and herbivore-resistant *Arabidopsis thaliana*. The leaves of 162 recombinant inbred lines (Rils) were screened to detect genotypes upon which *Plutella* larvae fed least (*P. xylostella*-resistant) or most (*P. xylostella*-susceptible). 2D-PAGE revealed significant differences in the proteomes between the identified resistant and susceptible Rils. The proteomic results, together with detection of increased production of hydrogen peroxide in resistant Rils, suggest a correlation between *P. xylostella* resistance and the production of increased levels of reactive oxygen species (ROS), in particular H_2_O_2_, and that this was expressed prior to herbivory. Many of the proteins that were more abundant in the *Plutella*-resistant Rils are known in other biological systems to be involved in limiting ROS damage. Such proteins included carbonic anhydrases, malate dehydrogenases, glutathione S-transferases, isocitrate dehydrogenase-like protein (R1), and lipoamide dehydrogenase. In addition, patterns of germin-like protein 3 isoforms could also be indicative of higher levels of reactive oxygen species in the resistant Rils. Consistent with the occurrence of greater oxidative stress in the resistant Rils is the observation of greater abundance in susceptible Rils of polypeptides of the photosynthetic oxygen-evolving complex, which are known to be damaged under oxidative stress. The combined results suggest that enhanced production of ROS may be a major pre-existing mechanism of *Plutella* resistance in *Arabidopsis*, but definitive corroboration of this requires much further work.

## Introduction

*Plutella xylostella*, the Diamondback Moth, is a specialist herbivore that feeds on species within the *Brassicaceae* family, including *Arabidopsis thaliana* [1]. *P. xylostella* is regarded as the most destructive insect pest of Brassicaceae crops throughout the world [2]. Yield losses occur through direct consumption and contamination of the harvested crop, affecting the cosmetic value of the crop [3]. In addition, expensive control measures add to its economic importance and *P. xylostella* has shown a remarkable capacity to develop resistance to chemical insecticides [4].

The *A. thaliana* - *P. xylostella* interaction is a model system used to investigate insect resistance in plants, in particular the analysis of inducible defence mechanisms [5]. Such studies have the advantage that comparisons can be made within genotypes before and after insect challenge (e.g. [6]), and hence the consequences of a change in the proteome can be measured. The current study has examined traits that are pre-existing prior to herbivory and are essentially constitutive (e.g. [7]). The analysis of pre-existing differences in the physiology of *A. thaliana* that influence insect resistance encounters some complications, including correlating similarities or differences between genotypes that relate to insect herbivory rather than to some other uncorrelated aspect of plant physiology/structure, and this is especially relevant for a proteomic study. There are many ecotypes of *A. thaliana* that differ widely in genotype and phenotype: as expected, this extends to differences in both protein spot expression and protein spots identified in root proteomes [8].

To investigate differences in feeding behaviour using proteomics, plants are required that display different phenotypes without having a large number of alleles at each gene. To achieve this, the resistance or susceptibility to *P. xylostella* herbivory of a population of Recombinant Inbred Lines (Rils), which was produced by crossing two distinct accessions of *A. thaliana*, one from the Cape Verde Islands (Cvi) and the other from Germany (Landsberg erecta, L*er*), was investigated by measuring the leaf area consumed. These Rils have been used previously and demonstrated to have a wide range of phenotypes (often more extreme than the parents) for many different characters [9]. Since the ecotypes have been inbred until near complete homozygosity, there will be usually only two (but a maximum of four) different allelic combinations at each gene segregating between the Rils derived from them, and so while we would expect each Ril to have a unique combination of polypeptides within its proteome, there will be strong protein similarities where phenotypic characters are shared. In this study, our purpose was to reveal those differences that correlate with *Plutella* larval feeding behaviour, and not the differences in the rest of the proteome. By pooling proteins from lines sharing resistance to herbivory, and comparing these to a pool of polypeptides from susceptible lines, we have attempted to dilute the differences between proteins within pooled samples and highlight spots that were common between individual proteomes within a pool (not all of which will be related to feeding behaviour). Such an approach has been described previously for transcriptomics using pooled RNA microarrays (e.g. [10]).

2D-PAGE, coupled with MS/MS, is one of the most established and effective techniques to undertake proteomic analysis, although it is less often used with plant leaves because of the difficulties encountered with the abundance of photosynthetic proteins which tend to mask other spots. Many plant proteomic studies have used roots or cell cultures for this reason, including the comparison of ecotypes described above [8], despite the fact that there are marked differences in the root and leaf proteomes [11]. In order to correlate differences in physiology to *P. xylostella*-resistance or susceptibility, we have used 2D-PAGE coupled with MS/MS to identify leaf proteins from *A. thaliana* Rils. Initially, we compared the proteomes of pooled *P. xylostella*-resistant Rils and pooled *P. xylostella*–susceptible Rils and identified 29 proteins that were differentially expressed, through MS/MS. We then proceeded to compare the proteomes of one individual *P. xylostella*-resistant and –susceptible Ril, and also examined whether leaf discs used in the larval feeding assay showed a different proteome to whole leaves.

## Methods

### Plant materials and culture

The *A. thaliana* Ril population (numbered 1–162) derived from the accession Landsberg *erecta* (L*er*) and the ecotype Cape Verde Islands (Cvi) were obtained from the Nottingham Arabidopsis Stock Centre. Plants were grown in a plant growth room at 22°C/18°C (day/night) and a photoperiod of 14 h light/10 h dark. The light intensity in the growth room was 70µE m^−2^ s^−1^ (Philips TLD36W/89). Plants were grown in 7cm wide pots containing a 3∶3∶1 mixture of John Innes No. 3, Levington MH and Silvaperl coarse pearlite. Seeds were applied to the soil surface, and underwent a cold stratification period at 4°C in the dark for 5 days, to ensure uniform germination. Seedlings were covered with propagator lids during the first week of germination. All experiments shown in this study were carried out on leaf numbers 3–6 taken from 4 week old plants. At this time point all plants were in the vegetative stage.

### Insect materials and culture

*P. xylostella* were obtained from Syngenta, Bracknell (Jealott's Hill International Research Centre), and reared at a temperature of 25°C and a humidity of approximately 60%. Insects were cultured in plastic containers (20×20×10cm) with holes in the lids to allow ventilation and netting to prevent escape. Adults were allowed to emerge from their pupal cases in the plastic containers, mate and lay eggs on creased parafilm covered in the juice from crushed cabbage leaves. The parafilm covered in eggs was removed every two days from the adult container and placed into fresh containers containing artificial diet (8% w/v wheatgerm, 3.5% w/v casein from bovine milk, 88mM sucrose, 2% w/v Agar, 1.5% w/v brewers yeast, 1% w/v Wesson salt mixture (Sigma), 18mM sorbic acid, 5mM cholesterol, 13mM methyl 4-hydroxybenzoate, 0.001% v/v boiled linseed oil, 1.2% w/v Vanderzant vitamin mixture (Sigma), 7mM choline chloride, 0.2mM formaldehyde). The larvae were left to hatch and develop in the same container without change of food, eventually forming pupae, which emerged and laid eggs onto fresh parafilm.

### Insect resistance screen

Plants were screened for resistance to *P. xylostella* herbivory in a no-choice leaf disc bioassay. 0.5cm^2^ leaf discs were cut from the centre of each leaf with a cork borer and placed adaxial surface upwards inside the wells of 24-well microtitre plates. Discs were kept moist through the addition of a 1% phytagel solution to the bottom of each well, prior to cutting of the disc. Two early second instar larvae were added to each leaf disc and left for 24 h. During the assay, special adhesive lids (AB Gene) were placed over the top of the 24-well plates to prevent larval well-to-well movement. Each assay took place under plant growth room conditions. Leaf area consumption was assessed through using a Win/Mac Folia leaf area meter (Regent Instruments Inc.).

### *In vivo* detection of H_2_O_2_ levels

Leaf wounding was achieved through piercing with a sharp pair of forceps. Leaf discs were taken in an identical manner to those used in the *P. xylostella* resistance assay. To assess H_2_O_2_ levels in *A. thaliana*, to provide evidence that the resistant plants produce more H_2_O_2_, leaf material was harvested and immediately vacuum-infiltrated with 0.1mg/ml 3,3′-diaminobenzidine (DAB)-HCl, pH 3.8 for 15 min. DAB staining was visualised following chlorophyll removal through boiling in ethanol. Darker staining in the leaves signifies greater levels of ROS.

For quantification of staining, discs and leaves were scanned with an Epson Perfection 3200 Pro colour scanner and images were transformed to black and white, with areas of leaves showing DAB staining being coloured black. The area, in pixels, of DAB stain was calculated using the Magic Wand Tool and Histogram function of Adobe Photoshop 6.0 (Adobe Systems, Mountain View, CA). The areas stained and areas of entire leaves, both in pixels, were used to calculate percentage areas of leaf stained [12]–[13]. For statistical analysis, the results were expressed as a proportion and the data were transformed using Arc Sine.

### Protein extraction

For proteomic comparison of *P. xylostella* -resistant and –susceptible Rils, three inbred lines (Resistant – Rils 28, 57 and 125; Susceptible = Rils 23, 49 and 162) each were combined for analysis. *A. thaliana* proteins were extracted using a modified version of the extraction procedure used for Fraction I in [14]. Leaf discs were harvested from *A. thaliana* plants and frozen immediately in liquid nitrogen. 300–1200mg of frozen plant material was used for extraction in microfuge tubes depending on the amount of protein to be loaded per gel. 100mg of tissue were extracted in each microfuge tube with the aid of a microfuge pestle. To each tube, 12.5µl of Mixture 1 (1× Complete™ protease inhibitor tablet [Roche] dissolved in 2ml of 100mM Potassium Chloride; 50mM Tris; 20% v/v Glycerol) and 5µl of Mixture 2 (1mM Pepstatin A; 1.4µM PMSF) were added. The homogenate was centrifuged at 20 000× *g* at 4°C for 30 min. The resulting supernatant was then centrifuged at 200 000× *g* at 4°C for 30 min. A quantity of the supernatant was precipitated overnight at −20°C in 5 times the volume of 10% TCA in acetone. The protein mixture was centrifuged at 6000× *g* for 5 min and the resulting pellet was washed in 200µl of an acetone solution containing 4mM PMSF, 2µM ethylenediaminetetraacetic (EDTA), and 0.07% v/v 2-Mercapto-ethanol. After centrifugation at 6000× *g* for 5 min, the supernatant was removed and the pellet was air dried for 5 min.

### 2D-PAGE and image analysis

For 2D-PAGE, IEF was performed using a Protean IEF cell (BioRad) and a 17cm ReadStrip with a linear pH gradient of 5 to 8 (BioRad). IPG strips were covered with mineral oil to prevent dehydration and were actively rehydrated at 50V for 12 h. Proteins (300 µg or 2,000µg for analytical and preparative gels, respectively; BioRad ‘2-D Electrophoresis for Proteomics: A Methods and Product Manual’, p7) in sample buffer (9M Urea, 4% 3-[(3-cholamidopropyl) dimethylammonio]-1-propanesulfonate (CHAPS), 1%ASB 14 detergent) were focused at 250V for 30 min (linear ramp), 10000V for 3 h, 10000 V to 40000V/h (linear ramp). IPG strip equilibration took place in equilibration buffer (50mM Tris (pH 6.8), 6M urea, 2% w/v SDS, 30% w/v glycerol, bromophenol blue) containing 20mM DTT for 15 min, followed by equilibration buffer containing 25mM IAA for 15 min. IPG strips were then loaded onto 12.5% SDS-PAGE gels and run at 15mA/gel until the dye from the agarose sealing gel had migrated into the resolving gel and then 30mA/gel until the dye front had run off the gel. Gels were stained either using the silver staining technique [15], or by Colloidal Coomassie staining [16].

The 2D-PAGE gels were scanned using a GS-710 Calibrated Imaging Densitometer (BioRad) and gel comparisons were performed using PDQuest version 7.31 (BioRad). Three biological replicates were carried out. The gel sets were normalised to overall gel staining density, with total gel density being the sum of the darkness of every pixel in the gel image. When a spot density was displayed, it was given as parts per million of the overall gel density, thus compensating for differences in protein loading and gel staining. A student's t-test was used to compare mean densities of protein spots, and spots differing by 2-fold expression or greater were selected.

### Protein digestion and identification

For identification of proteins by mass spectrometry, a sample (2mg protein) was run on 2D-PAGE, staining with Colloidal Coomassie and protein spots of interest were excised. Spots were matched to the silver-stained analytical gels manually by two independent persons. After de-staining of gel plugs by washing in 50% v/v acetonitrile, 50% v/v 50mM ammonium bicarbonate at 37°C until clear, they were dehydrated in 100% acetonitrile at 37°C. For enzyme digestion with trypsin, the gel plugs were rehydrated in 10µl of trypsin (0.1µg trypsin [Promega] in 10µl of 50mM ammonium bicarbonate), and incubated at 37°C overnight.

Enzyme digests were analysed using an UltiMate nano-liquid chromatograph (LC Packings) connected to a Waters (Manchester, UK) Q-TOF Micro electrospray tandem mass spectrometer, operated in positive ion mode. Chromatography was carried out on a μ-Precolumn C18 cartridge (LC Packings) connected to a PepMap C18 column (3µm 100Å packing; 15cm×75µm i.d.), using a linear gradient of 5% v/v solvent B (0.1% v/v formic acid in 80% v/v acetonitrile in water) in solvent A (0.1% v/v formic acid in 2% v/v acetonitrile in water) to 100% solvent B over 60 min at a flow rate of 200nl/min. The spectrometer was operated in Data Directed Analysis mode, where a survey scan was acquired over m/z 400–1500, with switching to MS/MS on multiply charged ions.

For the MS/MS data derived from doubly charged ions, peak lists were created using Masslynx (version 4); subtraction was performed with the following parameters: polynomial order 15, 50% below curve, tolerance 0.01. Smoothing of the peak list was performed using the Savitzky Golay method to 2 smooths. The data were centred using the centroid top method at 80%. MS/MS ion searches of the NCBI MSDB database restricted to the *A. thaliana* entries were undertaken using the MASCOT search engine version 2.2 (http://www.matrixscience.com) to yield protein identifications. Searches were performed without restriction of protein Mr or pI and were restricted to trypsin cleavage products, with one trypsin miscleavage being allowed. Searches did not take into account any fixed or variable modifications. Peptide mass tolerance and fragment mass tolerance were set to 2.0Da and ±0.8Da, respectively. Furthermore, partial peptide sequences were determined by manual interpretation of MS/MS data using the PepSeq software within the MassLynx package (Waters). The resulting sequences were then used to search the NCBI database using the BLAST algorithm and the option to search for short nearly exact matches (www.ncbi.nbm.nih.gov/BLAST/). In most cases, peptides were matched to proteins when statistically significant MASCOT probability scores (<0.05) were consistent with the protein experimental pI and Mr and when the manually-derived partial peptide sequences matched the database protein sequence. However, in a few cases, a statistically significant MASCOT probability score was not observed and the protein identification was based on matching of peptide sequences (generally two or more, but in a minority of cases, one sequence) with the database protein sequence, in addition to the pI and Mr. Where peptides matched more than one member of a protein family with a probability score <0.05, precise identifications were made when proteins matched the observed pI and Mr.

## Results and Discussion

### Proteomic comparison of *P. xylostella*-resistant and -susceptible Rils

Using an assay based on leaf consumption by *Plutella* larvae (Insect resistance screen), *A. thaliana* Rils and ecotypes were screened to identify differential resistance levels. Six replicate plants per Ril were used for each assay and four leaves per plant (leaves three to six). The size of the experiment made it impractical, both in terms of time and the number of larvae required, to perform all of the assays on the same day. Consequently, the plant lines were randomly selected and screened in batches on different days. However, for each assay a control Ril (Ril 94) was also assayed each time to account for possible inter-batch experimental differences. The results were expressed as a percentage of the feeding damage for this Ril and the data were analysed with a one-way ANOVA and a significant difference across the population tested was found (P<0.001, f = 7.57, d.f. 110) (see Fig. S1 in Supporting Information). Despite the results being expressed as a percentage, data transformation was not necessary as the data were inspected for normality and for the relationship between fitted values and residuals. With the exception of a few outliers, a normal probability plot of the residuals showed that the residuals were normally distributed. A series of 30 plant lines was selected for further assay, that displayed the extreme (the most and least damaged) and intermediate phenotypes . By using fewer *A. thaliana* lines, further analyses could be made within single experiments replicated at three different times, reducing possible variables arising between batches assayed on different days. Additionally, *P. xylostella* resistance could be expressed as leaf area consumed rather than as a percentage of the control Ril.

**Figure 1:**
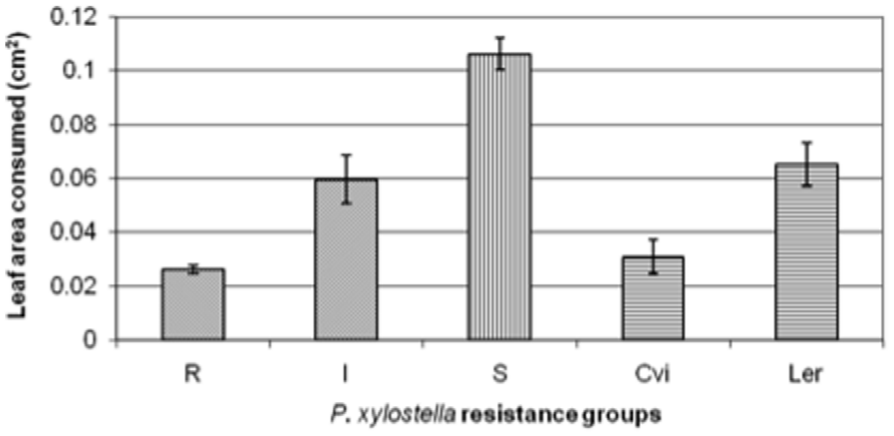
*A. thaliana* leaf area consumed by *P. xylostella* feeding on Rils (Recombinant Inbred Lines) and ecotypes of various ‘insect-resistance groups’. Mean leaf area consumed during a 24 h period is given for the R (Resistant), I (Intermediate), and S (Susceptible) Rils, plus their parents (Cvi and L*er*). Three R (Rils 28, 57, 125), three S( Rils 23, 49, 162), and one I Ril (Ril 93) were selected to represent the different ‘resistance groups’ across the Ril population. For each ecotype and I Ril, 6 plants were taken, each of which furnished 4 leaves, with a leaf disc produced from each leaf. Three replicates of such plants were done, thus, yielding 24×3 individual disc assays. In the case of R and S Rils, discs as above from 3 Rils were combined in each case to yield 72×3 individual assays. Values are means ± SEM for 3 individual experiments. A one-way ANOVA statistical analysis was undertaken and showed that there was a significant difference (p = 0.000, f = 15.05, d.f. = 4), between the different populations. The LSD (Least Significant Difference) was calculated and significant differences were found between R and I, R and S, I and S, Cvi and L*er*; neither R and Cvi nor I and L*er* were significantly different.

**Figure 2:**
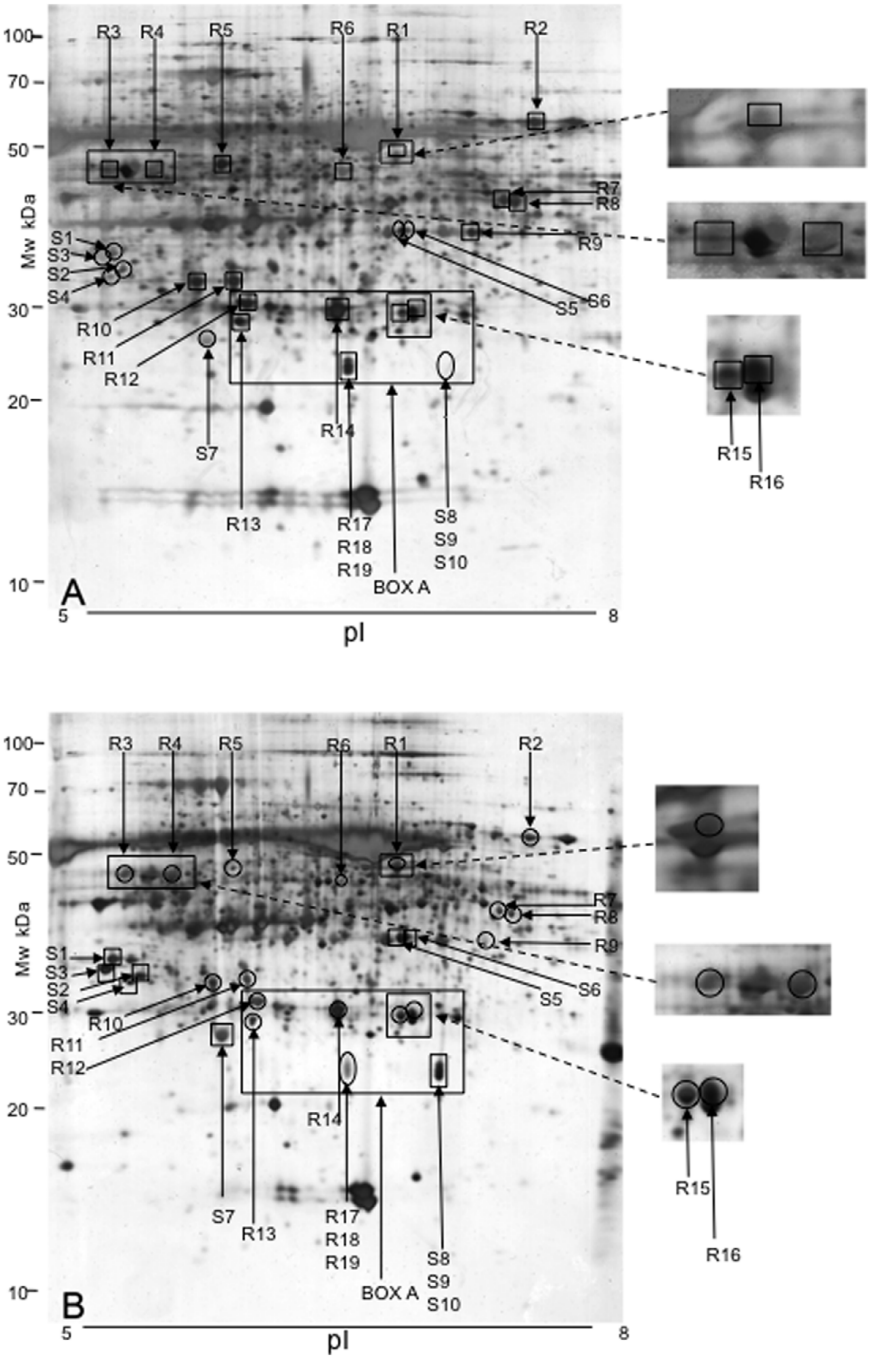
2D-PAGE images of protein extracts from leaf discs of *A. thaliana* plants. (A) Protein profile of pooled *P. xylostella*-resistant Rils (Recombinant Inbred Lines). (B) Protein profile of pooled *P. xylostella*-susceptible Rils. Enlargements of Box A are shown in Figure 3. Protein extracts were run on pH 5–8 IPG strips for IEF, and followed by SDS PAGE (12.5% PAGE). Protein spots marked with a box were more abundant on that gel, whereas ones marked with a circle were less abundant on that gel. For ‘R’ spots, the protein was more abundant in the ‘Resistant’ sample compared to the ‘Susceptible’ one. For the ‘S’ spots, the converse applied.

**Figure 3:**
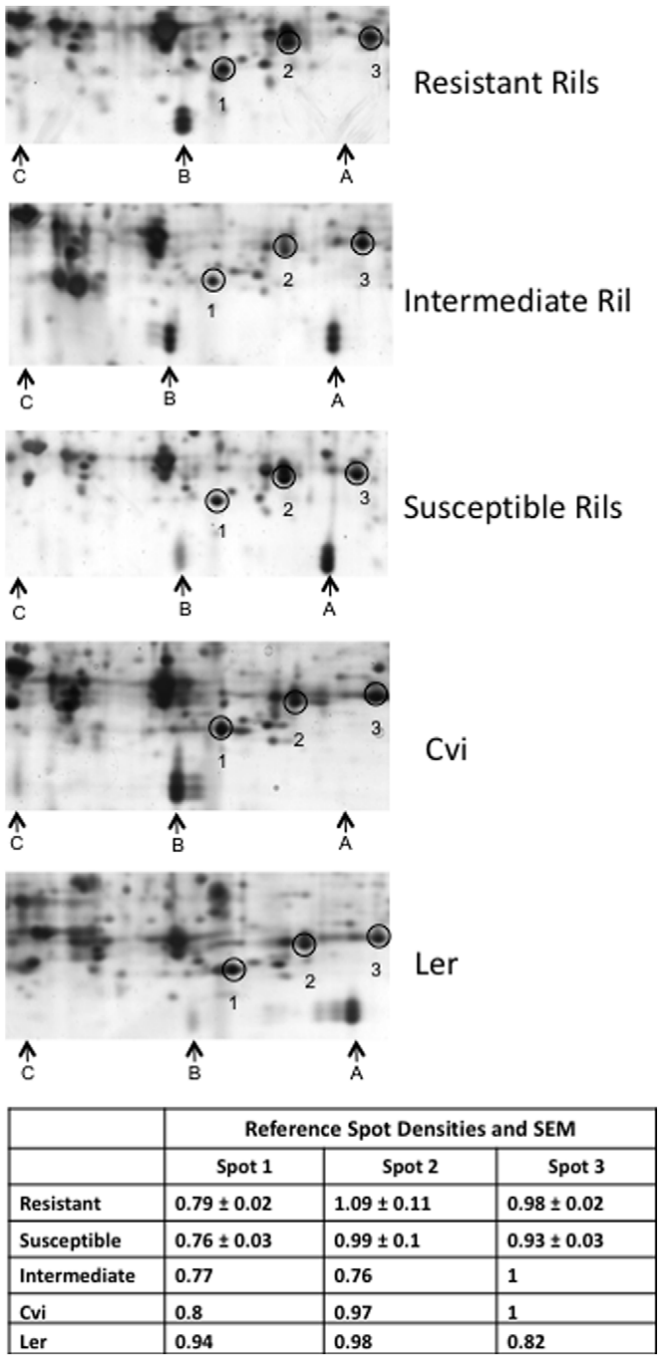
Enlargements of Box A from Fig. 2 (A) & (B), together with equivalent areas from 2D-PAGE gels of proteins from an intermediate resistance Ril and the parents (Cvi and Ler). These show the locations and intensities of GLP3 isoforms amongst the various *A. thaliana* lines studied. Internal reference proteins that do not change among the inbred lines and parental ecotypes are marked by circles. Results for the intensities of reference proteins in Resistant and Susceptible Rils are the means ± SEM of three biological replicates, whereas those for the Intermediate Ril and parental ecotypes are for single samples.

Due to their consistency throughout the biologically replicated assays, seven Rils, plus their parents were selected for further analysis. The seven chosen Rils were grouped into R, I, or S to signify their ‘resistance’ classification (i.e. R = resistant; I = intermediate; S = susceptible) (Figure 1). Insect feeding was approximately four times greater on the three S Rils compared to the three R Rils. In addition, insect feeding was two times greater on the L*er* accession compared to Cvi. This is contrary to what has been found in previous studies (e.g. [17]–[18]). Probable explanations include the use of different instars of larvae and assay times. Kliebenstein *et al.* [17] used first instar *P. xylostella* larvae, whilst second instar larvae were used in the current work. The biology of first and second instar larvae is likely to differ greatly (first instar larvae are leaf miners and second instar larvae are leaf chewers). The differential feeding by *P. xylostella* between plant genotypes resulted from physiological differences either in pre-existing expression of defence-related components or induced rapidly during the assay, perhaps as a result of cutting leaf discs: therefore, a comparison was made at the proteomic level. Pooled leaf material (unchallenged by insects) from the three R lines was compared by SDS-PAGE to that from the S genotypes (Figure 2) and a large number of protein spots showed differential expression: these are marked on Figure 2 (A) & (B), and identified proteins are shown in Table 1. In total, 50 protein spots were found to be more abundant in the resistant Rils, whilst 17 spots were expressed at greater levels in the susceptible Rils. The area represented by a box in Figure 2 (A) & (B) was analysed in more detail (Figure 3) and is discussed later (Further analysis of GLP3).

**Table 1:** Identity of differentially expressed proteins from pooled *P. xylostella*-resistant or –susceptible *A. thaliana* Rils.

Protein spot^a^	Protein identified	Gene	Matched peptides in database	Mascot MOWSE score	Sequence coverage %	Obs mass/pI (kDa)	Theo mass/pI (kDa)	Fold Difference^a^	t-test p-value
R1	Isocitrate dehydrogenase-like protein	At5g14590	NILDGTVFRDIFQEVYEANWK	66	4	48.3/6.6	52.0/7.1^b^	6.3	0.045
R2	Glycine hydroxymethyltransferase	At4g37930	EVLYDFEDKAYQEQVLSNSAK	68	4	52.0/7.5	57.4/8.1	4	0.006
R3	Glutamate-ammonia ligase precursor, chloroplast	At5g35630	TIEKPVEDPSELPKEEGGFEVIKAILNLSLR	113	7	48.6/5.0	47.3/6.4	c	0.05
R4	NADP-dependent malate dehydrogenase	At5g58330	SSAASTAVSIVDAIK	60	3	48.6/5.5	48.2/5.8	2.1	0.047
R5	Transketolase-like protein	At3g60750	KYPEEASELKSIITGELPAGWEKALPTYTPESPGDATRTPSILALSRLPHLPGTSIEGVEKESVLPSDVSARVSIEAASTFGWGKSIGINSFGASAPALLYKEFGITVEAVVDAAK	398	14	48.8/6.1	79.9/5.9	14.5	0.047
R6	Glyceraldehyde-3-phosphate dehydrogenase (NADP) (phosphorylating) B precursor	At1g42970	DSPLEVVVLNDSGGVKIVDNETISVDGKVLDEEFGIVKAAALNIVPTSTGAAK	228	13	47.2/6.4	47.7/6.3^b^	17.4	0.001
R7	Glyceraldehyde-3-phosphate dehydrogenase C subunit	At3g04120	AASFNIIPSSTGAAKGILGYTEDDVVSTDFVGDNR	185	10	43.7/7.2	43.5/7.1^b^	4.2	0.05

Protein spot^a^	Protein identified	Gene	Matched peptides in database	Mascot MOWSE score	Sequence coverage %	Obs mass/pI (kDa)	Theo mass/pI (kDa)	Fold Difference^a^	t-test p-value
R8	Glyceraldehyde-3-phosphate dehydrogenase C subunit	At3g04120	VPTVDVSVVDLTVRGILGYTEDDVVSTDFVGDNR	260	10	43.5/7.3	43.5/7.1^b^	4.4	0.05
R9	Mitochondrial NAD-dependent malate dehydrogenase OR NAD-dependent malate dehydrogenase	At1g53240 OR At3g15020^d^	ALEGADLVIIPAGVPRLFGVTTLDVVRTQDGGTEVVEAK	174	12	35.6/7.0	35.8/8.3	6.1	0.046
R10	Putative carbonic anhydrase	At5g14740	ITAELQAASSSDSKSFDPVERYETNPALYGELAKYAGVGAAIEYAVLHLKV	161	19	30.5/6.1	28.3/5.4^b^	5.8	0.044
R11	Putative carbonic anhydrase	At5g14740	ITAELQAASSSDSKSFDPVER	91	8	30.5/6.1	28.3/5.4^b^	3.5	0.042
R12	Probable triosephosphate isomerase	At2g21170	GGAFTGEISVEQLKGPEFATIVNSVTSK	109	9	28.6/6.1	33.3/7.7	6.1	0.05
R13	2, 4-D inducible glutathione S-transferase	At1g78370	GVEFEYRNPFFPSDPYGRFWADFVDKFTDAQFKFGNFSIESESPK	165	21	26.9/6.1	24.9/5.6	5.7	0.046
R14	Putative carbonic anhydrase, chloroplast precursor	At3g01500	AFDPVETIKYETNPALYGELAKYGGVGAAIEYAVLHLKEAVNVSLANLLTYPFVRGGYYDFVK	243	23	27.6/6.4	29.5/5.5	2.2	0.05
R15	Carbonic anhydrase, chloroplast	At3g01500	YGGVGAAIEYAVLHLKEAVNVSLANLLTYPFVR	141	23	26.9/6.7	29.5/5.5	2	0.05
R16	Glutathione S-transferase	At4g02520	YENQGTNLLQTDSK	43	6	27.2/6.7	24.1/6.3	2	0.046
R17	Germin-like protein 3	At5g20630	GDSMVFPQGLLHFQLNSG	43	9	22.8/6.5	21.8/6.8	7.9	0.023
R18	Germin-like protein 3	At5g20630	GDSMVFPQGLLHFQLNSG	43	9	22.2/6.5	21.8/6.8	8.2	0.019
R19	Germin-like protein 3	At5g20630	GDSMVFPQGLLHFQLNSG	43	9	21.8/6.5	21.8/6.8	7.8	0.012

Protein spot^a^	Protein identified	Gene	Matched peptides in database	Mascot MOWSE score	Sequence coverage %	Obs mass/pI (kDa)	Theo mass/pI (kDa)	Fold Difference^a^	t-test p-value
S1	Putative p-nitrophenylphosphatase	At5g36790	LVFVTNNSTKVYVIGEEGILKELELAGFQYLGGPDDGKIQPDFYTSKISDFLSPK	229	18	34.2/5.0	34.0/5.1	3.7	0.044
S2	33kDa polypeptide of oxygen-evolving complex (OEC) in Photosystem II	At5g66570	NAPPEFQNTKVPFLFTVKFLVPSYRGGSTGYDNAVALPAGGRGDEEELVKNTAASVGEITLK	257	19	33.4/5.0	35.2/5.6^b^	3.2	0.021
S3	33kDa polypeptide of oxygen-evolving complex (OEC) in Photosystem II	A0t5g66570	LTYDEIQSKNAPPEFQNTKGGSTGYDNAVALPAGGRNTAASVGEITLK	177	14	33.4/5.1	35.2/5.6^b^	7.1	0.045
S4	Photosystem II oxygen-evolving complex protein 1	At3g50820	LTYDEIQSKNAPPEFQNTKVPFLFTVKKFLVPSYRGSSFLDPKNTAASVGEITLK	267	21	31.7/5.5	35.2/5.6^b^	2	0.05
S5	Mitochondrial NAD-dependent malate dehydrogenase	At1g53240	ALEGADLVIIPAGVPRDDLFNINAGIVKLFGVTTLDVVRTQDGGTEVVEAKEGLEALKPELK	295	18	36.1/6.6	35.8/8.3	2.4	0.05
S6	Mitochondrial NAD-dependent malate dehydrogenase OR NAD-dependent malate dehydrogenase	At1g53240^e^ OR At3g15020^b^	ALEGADLVIIPAGVPRLFGVTTLDVVRTQDGGTEVVEAK	174	12	36.4/6.7	35.8/8.3	2.2	0.05

Protein spot^a^	Protein identified	Gene	Matched peptides in database	Mascot MOWSE score	Sequence coverage %	Obs mass/pI (kDa)	Theo mass/pI (kDa)	Fold Difference^a^	t-test p-value
S7	Oxygen-evolving 23kDa protein	At1g06680	TNTDFLPYNGDGFKEIEYPGQVLRHQLITATVNGGKKFVESAATSFSVA	181	19	24.4/6.0	28.1/6.9	5.3	0.043
S8	Germin-like protein 3	At5g20630	NPDQVTENDFAFTGLGK	f	8	22.7/6.8	21.8/6.8	g	0.001
S9	Germin-like protein 3	At5g20630	NPDQVTENDFAFTGLGK	f	8	22.2/6.8	21.8/6.8	g	0.001
S10	Germin-like protein 3	At5g20630	NPDQVTENDFAFTGLGK	f	8	21.8/6.8	21.8/6.8	g	0.001

2D-PAGE protein profiles from pooled *P. xylostella*-resistant or –susceptible *A. thaliana* Rils were compared and differentially expressed proteins identified. Spot numbers refer to Fig. 2. ‘Gene’ refers to the unique identifier in the *A. thaliana* database (www.arabidopsis.org). Identified proteins that were differentially expressed by 2-fold or greater are listed here. The fold difference represents the mean on the three gels using different biological samples (n = 3). P-value refers to the significance of the difference in intensity for each spot between R and S Rils following the application of a *t*-test.

a) For ‘R’ spots, protein was more abundant in the ‘Resistant’ sample compared to the ‘Susceptible’ one. For the ‘S’ spots, the converse applied.

b) Mass/pI gained from NCBI BLAST. All other mass/pI values came from TAIR or Mascot.

c) Protein spot absent from ‘Susceptible’ sample, thus fold difference cannot be given.

d) It was not possible to differentiate between these possible proteins by MS/MS, since only one peptide was detected in spots and this corresponds to a common region in the protein sequence. However, this is the likely correct identification – see ‘Results and Discussion’.

e) Likely correct identification – see ‘Results and Discussion’.

f) One peptide identified through manually sequencing the mass spectra.

g) Protein spot absent from ‘Resistant’ sample, thus fold difference cannot be given.

**Figure 4:**
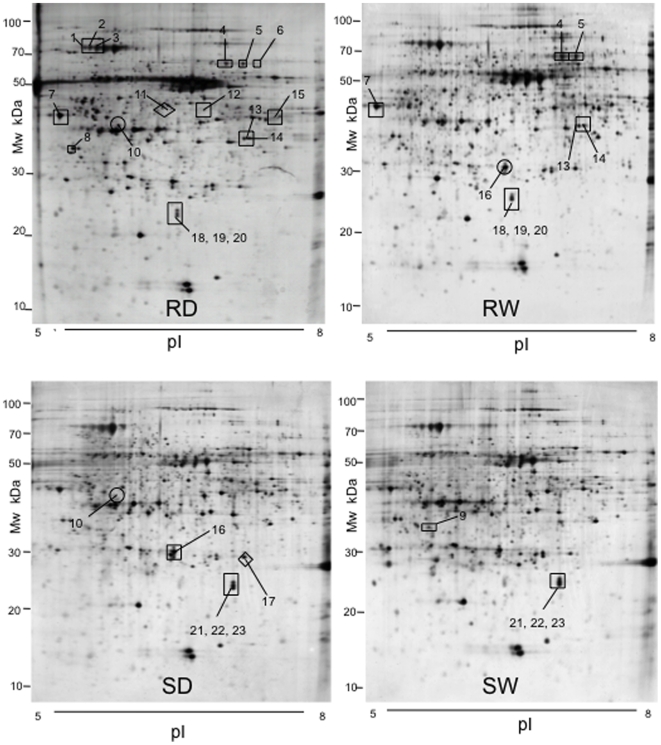
2D-PAGE images of protein extracts from leaf discs and whole leaves taken from *A. thaliana* plants. (RD) Protein profile of leaf disc samples taken from Ril (Recombinant Inbred Line) 57 (*P. xylostella*-resistant Ril). (RW) Protein profile of whole leaf samples taken from Ril 57 (*P. xylostella*-resistant Ril). (SD) Protein profile of leaf disc samples taken from Ril 23 (*P. xylostella*-susceptible Ril). (SW) Protein profile of whole leaf samples taken from Ril 23 (*P. xylostella*-susceptible Ril). Protein spots marked □ were more abundant in that particular Ril as compared to the other Ril when analysing the same leaf type; ○ were of increased abundance in discs or whole leaves within the same genotype; ◊ were more abundant in one particular set of gels, indicating specificity to both the genotype and leaf sampling method.

A similar proteomic analysis was then repeated with individual *P. xylostella*-resistant (Ril No. 57) and -susceptible (Ril No. 23) samples using leaf discs, but also using protein samples from whole leaves to examine whether there was a change in the proteome following cutting of the disc. This created a 4-way comparison: RD (resistant discs) vs. SD (susceptible discs); RD vs. RW (resistant whole leaves); RW vs. SW (susceptible whole leaves); SD vs. SW. Typical 2D-PAGE images are shown in Figure 4.

### Identification of differentially expressed proteins in pooled R and S Rils

The proteins that were differentially expressed in resistant and susceptible Rils were subjected to mass spectrometry for identification. Out of the 50 protein spots that were more abundant in the resistant Rils, 19 were successfully identified, whilst 10 out of the 17 protein spots of greater abundance in the susceptible Rils were identified (Table 1); many of the proteins that were not identified were of low abundance. Whilst differential expression of proteins in resistant and susceptible Rils suggests their involvement in the resistance mechanism, demonstration of their direct role requires much further experimentation. Importantly, a number of the identified proteins have been demonstrated in various other biological systems to have functions related to overcoming oxygen stress or reactive oxygen species.

Of the 19 spots that were more abundant in the resistant Rils, four were identified as carbonic anhydrases, two (R14 & R15) being products of the *A. thaliana* gene At3g01500, a chloroplast carbonic anhydrase; the other two (R10 & R11) were products of At5g14740, a putative carbonic anhydrase. Each of these pairs of carbonic anhydrases had a very similar mass and only differed slightly in their pI. In Yeast, deletion of the NCE103 gene led to significantly lower carbonic anhydrase levels and increased oxygen sensitivity compared to wild type control strains [19]. Furthermore, after transformation with carbonic anhydrase genes, normal growth was observed under aerobic conditions [20].

Three protein spots (R6, R7, & R8) were identified as glyceraldehyde-3-phosphate dehydrogenase or its precursor, (R6, At1g42970, chloroplast; R7 and R8, At3g04120, cytosolic).

Two malate dehydrogenases (R4 & R9) were more abundant in the resistant Rils, with R4 being NADP-dependent malate dehydrogenase (At5g58330). Database searching indicated that R9 was either mitochondrial NAD-dependent malate dehydrogenase (At1g53240) or NAD-dependent malate dehydrogenase (At3g15020), since it was impossible to distinguish between the latter two because their predicted peptide sequences are identical in the regions of the MS-sequenced peptides. However, since mitochondrial NAD-dependent malate dehydrogenase has been shown to be more abundant in susceptible Rils (see below), it is likely that R9 is NAD-dependent malate dehydrogenase. It has been shown that a mutant *Escherichia coli* strain, with reduced malate dehydrogenase activity, was more sensitive to H_2_O_2_ treatments compared to the wild type strain [21].

In addition to this, two glutathione S-transferases (R13 & R16) were upregulated in the resistant Rils, with R13 being annotated as a putative auxin-inducible glutathione S-transferase, and associated with limiting oxygen damage [22]. Glutathione S-transferases are commonly implicated in conferring resistance to many stresses including insecticide resistance in *P. xylostella* [23] and oxidative stress [24]–[25].

An isocitrate dehydrogenase-like protein (R1) was also observed to be more abundant in the R Rils, and is known to be involved in resistance to oxidative Stress [26]–[28]. The other protein spots identified to be abundant in the resistant Rils consisted of a mitochondrial glycine hydroxymethyltransferase (R2), a chloroplastic glutamate-ammonia ligase precursor (R3), a transketolase-like protein (R5), and a triosephosphate isomerase (R12).

Four of the identified spots (S2, S3, S4, & S7) that were more abundant in the susceptible Rils compared to the resistant Rils, were prominent members of the oxygen-evolving complex (OEC) from Photosystem II: both S2 and S3 were identified as the 33kDa polypeptide of the OEC; S4, the photosystem II OEC protein 1; S7, the 23kDa polypeptide. The OEC is responsible for the oxidation of water, allowing photosystem II to convert light to chemical energy, and is composed of subunits bound to the lumen side of photosystem II [29]. It has been well documented that high levels of ROS damage the OEC subunits resulting in degradation [30], as has possibly been observed in this study in the R Rils. Degradation of the OEC 33 and 23 subunits has been demonstrated upon strong light illumination, which was associated with raised ROS levels [29], [31]–[32]. If raised levels of ROS do impair the resistant Rils' photosynthetic capability, this might represent a cost to the plant. Such a cost may involve growth suppression such as that reported in *A. thaliana* plants with a cytosolic ascorbate peroxidise gene knocked out [33]. Such a potential cost would have to be weighed up against other roles of raised ROS such as initiating signal transduction pathways for pathogen defence [34] or acclimatisation to extreme environments [35] before deciding whether such plants could exist outside of a laboratory environment.

Two malate dehydrogenases (S5 & S6) were identified as being more abundant in the susceptible Rils, with S5 being mitochondrial NAD-dependent malate dehydrogenase, and database searches of S6 indicating either the same protein or NAD-dependent malate dehydrogenase. Since NAD-dependent malate dehydrogenase is more abundant in R Rils (above), it is likely that S6 is mitochondrial NAD-dependent malate dehydrogenase.

### Identification of differentially expressed proteins from individual genotypes

For comparison, the proteomes of two individual Rils were also analysed, the resistant Ril 57 and susceptible Ril 23 lines; this also enabled the assessment of the impact of cutting leaf discs relative to whole leaves (Figure 4).

In total, 23 protein spots were shown to differ between the 2D-PAGE gel comparisons (see Table 2). However, the differences between genotypes (R vs. S) proved more significant than between leaf sampling (W vs. D).

**Table 2:** Identity of differentially expressed proteins from whole leaves or leaf discs from individual *P. xylostella*-resistant (Ril57) or –susceptible (Ril23) *A. thaliana* Rils.

Protein spot	Protein name	Gene	Matched peptides in database	MOWSE score	Sequence coverage %	Obs mass/pI	Theo mass/pI	Fold difference	p-value
1	Thioglucoside glucohydrolase	At5g26000	LPEFSETEAALVK	58	2	62.2/5.7	61.1/5.7	RD vs. SD = 8.9	RD vs. SD = 0.028
2	Thioglucoside glucohydrolase	At5g26000	LPEFSETEAALVK	58	2	62.2/5.8	61.1/5.7	RD vs. SD = 5.7	RD vs. SD = 0.002
3	Thioglucoside glucohydrolase	At5g26000	YYNGLIDGLVAKLPEFSETEAALVK	78	5	62.2/5.9	61.1/5.7	RD vs. SD = 2.5	RD vs. SD = 0.035
4	Lipoamide dehydrogenase precursor	At3g17240	IVSSTGALSLTEIPKTPFTSGLDLEKVPGVVYTYPEVASVGK	128	8	57.1/7.0	54.0/7.0	RD vs. SD = a, RW vs. SW = a	RD vs. SD = 0.005, RW vs. SW = 0.007
5	Lipoamide dehydrogenase precursor OR Lipoamide dehydrogenase 1	At3g17240 OR At1g48030^b^	TPFTSGLDLEK	50	2	57.1/7.2	54.0/7.0 OR 54.0/7.5	RD vs. SD = a, RW vs. SW = a	RD vs. SD = 0.001, RW vs. SW = 0.018
6	Lipoamide dehydrogenase precursor OR Lipoamide dehydrogenase 1	At3g17240 OR At1g48030^b^	TPFTSGLDLEK	49	2	57.0/7.4	54.0/7.0 OR 54.0/7.5	RD vs. SD = a	RD vs. SD = 0.017
7	Sedoheptulose-bisphosphatase	At3g55800	FEETLYGTSR	37	2	44.2/5.0	42.4/6.5	RD vs. SD = 13.7, RW vs. SW = 6.8	RD vs. SD = 0.000, RW vs. SW = 0.000

Protein spot	Protein name	Gene	Matched peptides in database	MOWSE score	Sequence coverage %	Obs mass/pI	Theo mass/pI	Fold difference	p-value
8	Putative p-nitrophenylphosphatase	At5g36790	LIEGVPETLDMLRLFGQDNGGCK	50^c^	4 or 7	34.2/5.1	34.0/5.1	RD vs. SD = 2.6	RD vs. SD = 0.033
9	Glyoxalase I, putative	At1g67280	TVIAFIEDPDGYK	61	3	33.6/6.0	39.2/7.0	SW vs. RW = a	SW vs. RW = 0.048
10	Coprogen oxidase, putative	At1g03475	PAYIPIVEKIESILVSLPLSAR	65^c^	7	36.5/6.1	34.2/5.6	RD vs. RW = a, SD vs. SW = a	RD vs. RW = 0.036, SD vs. SW = 0.000
11	Glutamate-1-semialdehyde aminotransferase	At3g48730	AGSGVATLGLPDSPGVPKLSQPGTYEYLDK	79	6	47.7/6.4	50.1/7.0	RD vs. SD = a	RD vs. SD = 0.000
12	Isocitrate dehydrogenase, putative	At1g65930	DTYLNTEEFIDAVAAELK	63	4	46.9/6.6	45.7/6.5	RD vs. SD = a	RD vs. SD = 0.001
13	Mitochondrial NAD-dependent malate dehydrogenase	At1g53240	SEVVGYMGDDNLAKALEGADLVIIPAGVPREGLEALKPELK	179	12	35.6/7.2	35.8/8.3	RD vs. SD = a, RW vs. SW = a	RD vs. SD = 0.012, RW vs. SW = 0.024
14	Mitochondrial NAD-dependent malate dehydrogenase OR NAD-dependent malate dehydrogenase	At1g53240 OR At3g15020^b^	DDLFNINAGIVK	43	3	35.7/7.3	35.8/8.3	RD vs. SD = a, RW vs. SW = a	RD vs. SD = 0.042, RW vs. SW = 0.035
15	Glyceraldehyde-3-phosphate dehydrogenase	At1g13440	NPEDIPWGEAGADFVVESTGVFTDKVPTVDVSVVDLTVR	192	12	44.1/7.6	36.9/7.2	RD vs. SD = a	RD vs. SD = 0.001
16	Putative carbonic anhydrase, chloroplast precursor OR Putative carbonic anhydrase	At3g01500 OR At5g14740^b^	YETNPALYGELAK	51	4 or 5	27.6/6.5	29.5/5.5 OR 28.3/5.4^d^	SD vs. RD = 2.3, RW vs. RD = 2.2	SD vs. RD = 0.02, RW vs. RD = 0.011

Protein spot	Protein name	Gene	Matched peptides in database	MOWSE score	Sequence coverage %	Obs mass/pI	Theo mass/pI	Fold difference	p-value
17	Glutathione S-transferase	At2g30860	QPAYLALQPFGTVPAVVDGDYK	75	10	26.9/7.1	24.1/7.0	SD vs. SW = 2.1, SD vs. RD = 2.9	SD vs. SW = 0.014, SD vs. RD = 0.003
18	Germin-like protein 3	At5g20630	GDSMVFPQGLLHFQLNSG	43	9	22.8/6.6	21.8/6.8	RD vs. SD = 7.7, RW vs. SW = 8.1	RD vs. SD = 0.019, RW vs. SW = 0.009
19	Germin-like protein 3	At5g20630	GDSMVFPQGLLHFQLNSG	43	9	22.5/6.6	21.8/6.8	RD vs. SD = 7.9, RW vs. SW = 7.7	RD vs. SD = 0.02, RW vs. SW = 0.011
20	Germin-like protein 3	At5g20630	GDSMVFPQGLLHFQLNSG	43	9	22.1/6.6	21.8/6.8	RD vs. SD = 7.7, RW vs. SW = 7.2	RD vs. SD = 0.014, RW vs. SW = 0.03
21	Germin-like protein 3	At5g20630	NPDQVTENDFAFTGLGK	c	8	22.9/7.1	21.8/6.8	RD vs. SD = a, RW vs. SW = a	0.001
22	Germin-like protein 3	At5g20630	NPDQVTENDFAFTGLGK	c	8	22.5/7.1	21.8/6.8	RD vs. SD = a, RW vs. SW = a	0.001
23	Germin-like protein 3	At5g20630	NPDQVTENDFAFTGLGK	c	8	22.1/7.1	21.8/6.8	RD vs. SD = a, RW vs. SW = a	0.001

2D-PAGE protein profiles from whole leaf or leaf discs samples taken from individual *P. xylostella*-resistant or –susceptible *A. thaliana* Rils were compared and differentially expressed proteins identified. Spot numbers refer to Fig. 4. ‘Gene’ refers to the unique identifier in the *A. thaliana* database (www.arabidopsis.org). Identified proteins that were differentially expressed by 2-fold or greater are listed here. The fold difference represents the mean on the three gels using different biological samples (n = 3). P-value refers to the significance of the difference in intensity for each spot between the different gel comparisons following the application of a *t*-test.

a) Protein spot absent from one sample, thus fold difference cannot be given.

b) It was not possible to differentiate between these possible proteins, since only one peptide was detected in spots and this corresponds to a common region in the protein sequence.

c) One peptide identified through manually sequencing the mass spectra.

d) Mass/pI gained from NCBI BLAST. All other mass/pI values came from TAIR of Mascot.

A series of proteins, or their isoforms, were more abundant in both the pooled and individual R Rils: malate dehydrogenase protein (At1g53240 or At3g15020), one spot which was present in the pooled Rils and two that were present in the individual Ril; isocitrate dehydrogenase (pooled Rils = At5g14590; individual Ril = At1g65930), malate dehydrogenase (pooled Rils = At5g58330; individual Ril = At5g53240 and At5g53240 or At3g15020), and glyceraldehyde-3-phosphate dehydrogenase (pooled Rils = At1g42970 and At3g04120; individual Ril = At1g13440). The relevance of these has been discussed above.

A number of other protein differences between Rils 57 and 23 may be of relevance to *P. xylostella* resistance. Three isoforms of lipoamide dehydrogenase were also more abundant in the R Ril, for which an antioxidant role has been identified previously [36]–[37]. Interestingly, three isoforms of thioglucoside glucohydrolase were also more abundant in the R Ril. These proteins, more commonly called myrosinases, are an essential component of the glucosinolate-myrosinase system of plant defence [38], and are known to be important in defence of *A. thaliana* against herbivory by several insect species [39]–[40].

Amongst the other proteins more abundant in the R Ril is sedoheptulose-bisphosphatase, a Calvin cycle enzyme found to be upregulated in resistant *B. curinata* plants upon inoculation with the fungal pathogen *L. maculans* [41]. The authors suggested that the resistant plants may possess a higher photosynthetic capacity which may contribute to the overall ability of the plants to ward off infection.

Some commonly reported antioxidant genes, including ascorbate peroxidase, catalase and superoxide dismutase were not observed in increased abundance in the R plants at the protein level. It is likely that a number of proteins of interest were either masked by proteins of greater abundance or were outside the isoelectric range of the gels used. It is also possible that such proteins did differ in expression between the R and S plants, however, were amongst a number of proteins that were not of high enough abundance to be identified by MS/MS in this study.

### Further analysis of GLP3

A series of protein spot triplets (Table 1: R17, R18, & R19; S8, S9, S10; Table 2: spots 18–23) were identified as various isoforms of germin-like protein 3 (GLP3). Each triplet contains three proteins with identical pI and only slightly different masses. The triplets R17/19 and S8/10 have identical masses but differ in their pI. A third potential triplet of identical mass was also present in R Rils. These protein isoforms were studied further by analysing proteins from R, I (Intermediate), and S Rils, as well as the parents, Cvi and L*er* (Figure 3). For convenience, the most basic triplet (S8, S9, & S10) is labelled A, with the middle triplet (R17, R18, & R19) labelled B, and the most acidic triplet labelled C. Triplets A and B were confirmed to be GLP3 by LC MS/MS, however, triplet C was too low in abundance to be identified. Although triplet C cannot be definitively identified, judging by its mass and pI relative to the other two triplets, it appeared possible that it was a third GLP3 triplet. Both triplets A and B were found in the susceptible Rils, with triplet A being the most abundant, whilst triplet B was comparatively much fainter. Triplet C was not observed in the susceptible Rils. In the resistant Rils, triplet B was much more abundant, with triplet A absent, and triplet C very faint. Identical results to those of the pooled samples were gained for each individual resistant and susceptible Ril (data not shown). The resistant Cvi showed a similar GLP3 isoform pattern to the resistant Rils, whilst the 2D-PAGE gel for L*er* was most similar to the susceptible Rils. The GLP3 isoform patterns for the intermediate Ril were also intermediate between the resistant and susceptible Rils with moderate levels of both triplets A and B present. Triplet C was absent from the intermediate Ril. As shown in Figure 3, the intensities of reference spots (circled) were essentially unchanged in the various gels, confirming that the differences in amounts of GLP3 isoforms are not due to differences in sample load. The presence of GLP3 in the form of triplets (Figure 3) has not previously been shown in any species to date.

GLP3 has been observed previously as doublets in both Col-0 and Ws-2 ecotypes of *A. thaliana*, and explained as arising by glycosylation [42]. Furthermore, that study showed that only one doublet was present per ecotype, each ecotype having a protein with a different pI arising from a single amino acid substitution. However, there is no evidence of two GLP3 genes in the *A. thaliana* database, and so it is likely that the GLP3 isoforms observed in this study result from post-translational modification. One possible modification that causes a shift in protein pI, but not mass, is the overoxidation of cysteines [43]–[46]. Unfortunately, an LC MS/MS analysis of peptide digests from triplets A and B could not corroborate such a modification in the GLP3 proteins. There does however remain a possibility that gene duplications could account for the presence of three GLP 3 triplets in other accessions such as L*er* or Cvi, which have not been completely sequenced.

The potential of GLPs in resistance to biotic stresses has been well documented [47]–[52]. A number of GLPs are known to have oxalate oxidase activity, an enzyme that degrades oxalate to carbon dioxide and H_2_O_2_ [47]. Of significance to insect resistance, cotton, transformed with a wheat germin gene, previously shown to confer oxalate oxidase activity, has demonstrated increased levels of resistance to the European corn borer [52]. Whilst H_2_O_2_ levels were greater in the transformed plants compared to the controls, feeding damage was reduced significantly. Although the resistance mechanism conferred by the wheat germin gene is uncertain, a number of possibilities have been suggested, including the direct effects of increased levels of H_2_O_2_ on the insect herbivore, increased signalling leading to the upregulation of certain defence genes, and the modification of plant cell wall chemistry, decreasing the palatability of the transformed plants. It has not been established whether *A. thaliana* GLPs possess oxalate oxidase or a related activity [53]. If the *A. thaliana* GLP3s are involved in an insect resistance mechanism, amounts of triplet B correlate with the degree of resistance in the Rils (Fig. 3).

### Analysis of leaf ROS levels and resistance to herbivory

**Figure 5:**
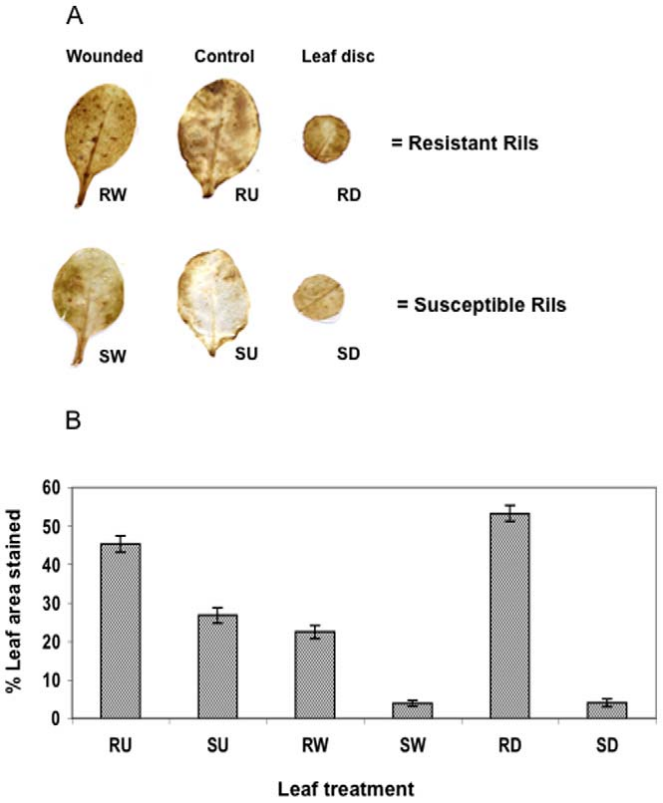
DAB staining to detect the presence of ROS in whole leaves and leaf discs of *A. thaliana* plants. (RW) Wounded whole leaf from an R Ril (Recombinant Inbred Line). (RU) Control, unharmed, whole leaf from an R Ril. (RD) Leaf disc taken from an R Ril. (SW) Wounded whole leaf from an S Ril. (SU) Control, unharmed, whole leaf from an S Ril. (SD) Leaf disc taken from an S Ril. Darker staining in the leaves signifies greater levels of ROS. For each experiment, four leaves per plant were used and triplicate experiments were done. (A) Representative samples of DAB staining, (B) Quantification of leaf areas stained. RU vs. SU, p = 0.000; RW vs. SW, p = 0.000; RD vs. SD, p = 0.000.

The results of the proteomic analysis suggested a possible correlation between the R Rils and raised levels of proteins with a potential antioxidant function. To investigate the oxidative state of leaves of the Rils further, the levels of ROS were examined by DAB staining. Figure 5 illustrates DAB staining in various leaf treatments taken from R and S Rils, with the darker stain in the R Rils, indicating consistently higher levels of ROS. In addition, ROS levels were increased at the locations of wounding, and at the edges of the leaf discs. Whilst in the R Rils, ROS levels were consistently high, in S leaves, the levels were low and only slightly raised after wounding. Possible roles of ROS in resistance to insects are unclear, and possibilities include direct toxicity to the insect gut, or acting as a signalling molecule that results in the upregulation of defence genes [52]. The role of ROS in response to wounding and insect herbivory has been well documented in other systems [54]–[62]. The situation may well be analogous in our current system. In a transcriptomic analysis of *P. xylostella* herbivory on L*er* plants, a number of genes involved in ROS production/breakdown were found to be induced [6]. Four methionine sulfoxide reducatase enzymes (At5g07460, At5g07470, At4g21840, At4g21850) whose function is to repair oxidatively damaged proteins (e.g. [63]–[64]) were upregulated during the 24 hour feeding period. Two thioredoxins and three glutaredoxins (e.g. [65]) were also induced by herbivory. Other genes that showed altered expression included peroxidises (e.g. [66]) (11 induced, 3 suppressed), glutathione S-transferases (e.g. [22]) (14 induced, 2 suppressed), ascorbate peroxidases/oxidases/reductases (e.g. [33]) (5 induced, 1 suppressed), and oxidoreductases (e.g. [67]) (16 induced, 4 suppressed). The expression of various catalase or superoxide dismutase genes was unaltered. The present proteomic study and transcriptomic analysis addressed above appear to correlate ROS with herbivory by *P. xylostella*. However, the two studies are clearly very different in that, here, the differences are pre-existing as the proteome was analysed in plants that were not subjected to herbivory, whilst the transcriptomic study examined the induction and suppression of genes in response to herbivory. When traits are measured in the absence of herbivore feeding [68]–[69], as in this study, it is always possible that the differentially expressed proteins could be upregulated or downregulated following the onset of herbivory. Of course, induced responses will also have an impact upon herbivory.

However, increased ROS production also requires protection of the plant's own physiology. It has been demonstrated from studies on other organisms, that glyceraldehyde-3-phosphate dehydrogenase can be modified through the overoxidation of its cysteine residues [70]. This enzyme, although an essential enzyme in the glycolytic pathway, has been located in parts of the plant cell not associated with its main function, such as the peribacteroid membrane [71] and the cell walls [72]. In *A. thaliana*, glyceraldehyde-3-phosphate dehydrogenase is a target of H_2_O_2_, and following H_2_O_2_ treatment, a reversible inhibition of its primary activity has been demonstrated [73]. In mammalian cells, overoxidation of a glyceraldehyde-3-phosphate dehydrogenase cysteine residue resulted in both a loss in the activity of its primary function, plus a new protein-protein interaction function allowing the protein to activate phospholipase D, and thereby initiating signalling [74]. Since three forms of glyceraldehyde-3-phosphate dehydrogenase were more abundant in the R Rils, it is possible that these together with GLP3 could act as a sink for ROS, through the overoxidation of cysteine residues [74], to protect other cellular components from the enhanced levels of ROS in R genotypes.

### Concluding remarks

In this study, we have demonstrated that proteomics by 2D-PAGE coupled with MS/MS, can be used to investigate complex phenotypes in a notoriously difficult plant organ, namely leaves. Furthermore, we have established that pooled samples can be used just as successfully as has been shown for transcriptomics.

After examining the herbivory of *Plutella* larvae on recombinant inbred lines (Rils) of *A. thaliana* to identify resistant and susceptible genotypes, the global analysis of their leaf proteomes has revealed consistent differences between them. The evidence from these proteomic observations and DAB staining suggests that the herbivory of *P. xylostella* larvae was lower in plants having increased levels of ROS. Although the roles of ROS in resistance to insects are unclear, possible mechanisms include direct toxicity to the insect gut, or they may act as signalling molecules that result in the upregulation of defence genes. Significantly, nineteen proteins were identified that were more abundant in R plants than in S plants, of which fifteen have been previously implicated in an anti-oxidative role (including carbonic anhydrase, malate dehydrogenases, glutathione S-transferases, isocitrate dehydrogenase-like protein, lipoamide dehydrogenase, glyceraldehyde -3-phosphate dehydrogenase), or involved in the production of hydrogen peroxide (e.g. GLPs). It is possible that glyceraldehyde-3-phosphate dehydrogenase and GLPs could act as a sink for ROS, through the overoxidation of cysteine residues, to protect other plant cellular components from the enhanced levels of ROS in the R genotypes. In contrast, amongst the proteins that were more abundant in the susceptible Rils compared to the resistant Rils were prominent members of the Photosystem II oxygen-evolving complex (OEC), which are known to be damaged under oxidative stress. This would likely result in their degradation in the resistant Rils.

In conclusion, while we cannot absolutely define enhanced production of ROS as a major pre-existing mechanism of *Plutella* resistance in *Arabidopsis*, there is a clear correlation between the higher levels of ROS, raised levels of antioxidant-associated proteins, reduced abundance of proteins known to be sensitive to ROS, and reduced feeding by *Plutella* larvae. Definitive establishment of such a role for ROS clearly requires much further work.

## Supporting Information

Figure S1 Mean feeding damage over a 24 hour period to A. thaliana lines by P. xylostella herbivory. For each A. thaliana line, 6 plants were taken, each of which furnished 4 leaves (leaves 3–6), with a leaf disc produced from each leaf. Due to the large number of lines to be assessed, they were screened in batches of 30 and the results were expressed as a percentage of a control Ril (Ril 94) which was challenged in each batch. This accounts for possible inter-batch experimental differences. The data were analysed with a one-way ANOVA and a significant difference across the population tested was found (P<0.001, f = 7.57, d.f. 110). Despite the results being expressed as a percentage, data transformation was not necessary as the data were inspected for normality and for the relationship between fitted values and residuals. With the exception of a few outliers, a normal probability plot of the residuals showed that the residuals were normally distributed. Not all of the population of 162 Rils could be assayed due to poor germination rates or the leaf size being too small.
(0.68 MB TIF)
