# Supplementary figures and images for: Differential Proteomic Analysis of Arabidopsis thaliana Genotypes Exhibiting Resistance or Susceptibility to the Insect Herbivore, Plutella xylostella
Source: PLoS One. 2010 Apr 8;5(4):e10103. doi: 10.1371/journal.pone.0010103 (PMC2851655; doi:10.1371/journal.pone.0010103)

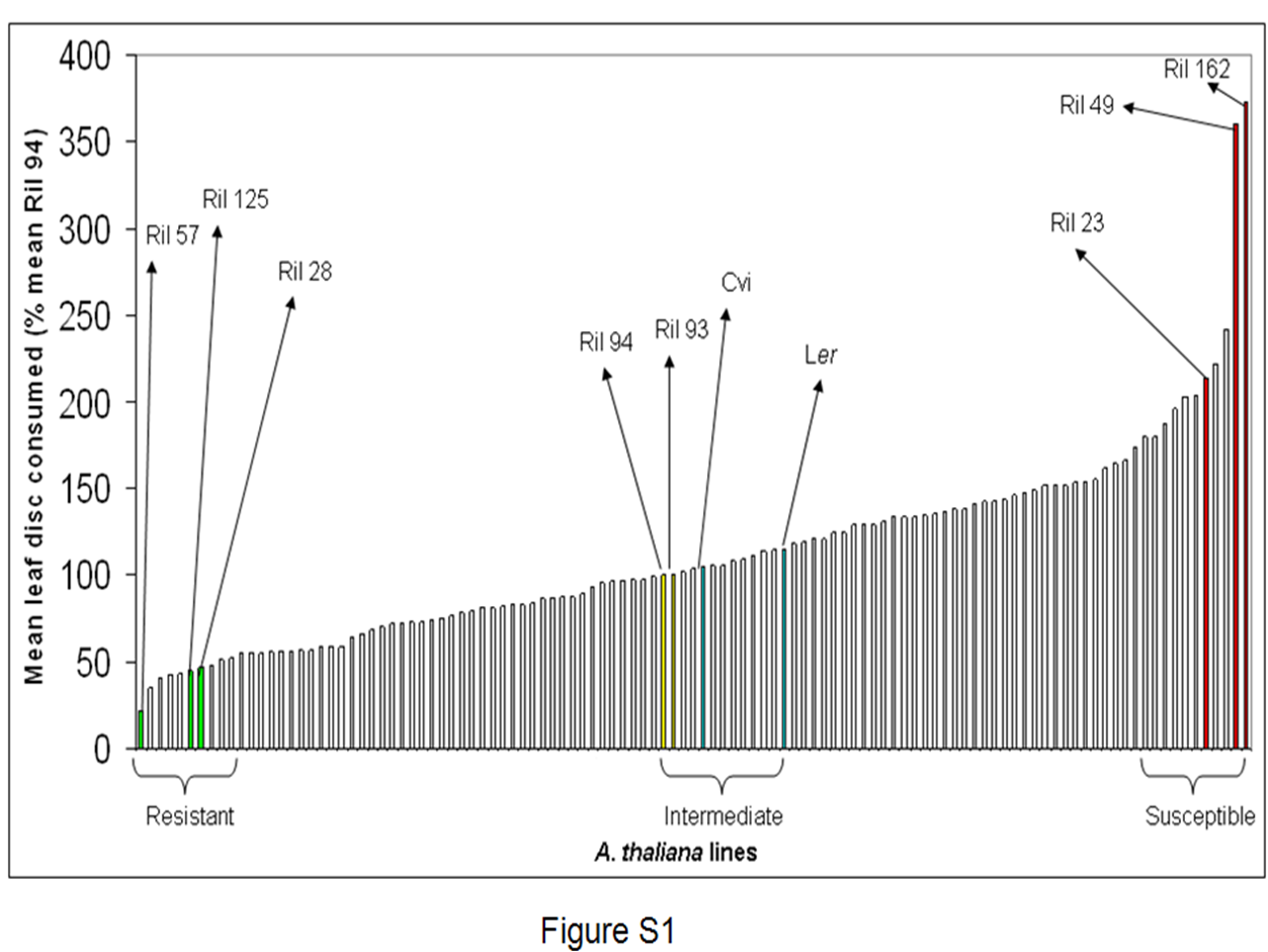

Supplement: Figure S1 — Mean feeding damage over a 24 hour period to A. thaliana lines by P. xylostella herbivory. For each A. thaliana line, 6 plants were taken, each of which furnished 4 leaves (leaves 3–6), with a leaf disc produced from each leaf. Due to the large number of lines to be assessed, they were screened in batches of 30 and the results were expressed as a percentage of a control Ril (Ril 94) which was challenged in each batch. This accounts for possible inter-batch experimental differences. The data were analysed with a one-way ANOVA and a significant difference across the population tested was found (P<0.001, f = 7.57, d.f. 110). Despite the results being expressed as a percentage, data transformation was not necessary as the data were inspected for normality and for the relationship between fitted values and residuals. With the exception of a few outliers, a normal probability plot of the residuals showed that the residuals were normally distributed. Not all of the population of 162 Rils could be assayed due to poor germination rates or the leaf size being too small. (0.68 MB TIF) [file pone.0010103.s001.tif]
